# Light Guided *In-vivo* Activation of Innate Immune Cells with Photocaged TLR 2/6 Agonist

**DOI:** 10.1038/s41598-017-08520-x

**Published:** 2017-08-14

**Authors:** Keun Ah Ryu, Bethany McGonnigal, Troy Moore, Tawnya Kargupta, Rock J. Mancini, Aaron P. Esser-Kahn

**Affiliations:** Department of Chemistry, University of California, Irvine, Irvine, CA 92697 USA

## Abstract

The complexity of the immune system creates challenges in exploring its importance and robustness. To date, there have been few techniques developed to manipulate individual components of the immune system in an *in vivo* environment. Here we show a light-based dendritic cell (DC) activation allowing spatial and temporal control of immune activation *in vivo*. Additionally, we show time dependent changes in RNA profiles of the draining lymph node, suggesting a change in cell profile following DC migration and indicating that the cells migrating have been activated towards antigen presentation.

## Introduction

Harnessing the innate and adaptive immune response has led to the development of vaccines and therapeutics^1–3^ . However, as the immune system “rivals the nervous system in complexity^4^,” understanding how to design better responses and therapies remains a challenge. One area of complexity is the presentation of antigens by the innate system to the adaptive system – including chemical signaling, spatial migration and cell-cell signaling. During this process, dendritic cells (DCs), activated by Toll-like receptors (TLRs) convey pathogenic information to the cells of the adaptive immune systems through the production of cytokines and cell surface markers^5, 6^. This process involves the migration of activated DCs into lymphatics to present antigens to T-cells^7–10^. However, understanding this complex system by manipulating sets of cells within it has been a challenge. Chemical control of various innate and adaptive immune cellular processes has been a burgeoning area of interest^11–16^. Recently, we developed a method to tag and remotely induce a guided immune response (TRIGIR) with a photo-caged TLR2/6 agonist^17^. TRIGIR allows for selective labeling of cells, followed by remote light activation. Here we use the TRIGIR method for *in vivo* light-based activation to control the migration of dendritic cells. We validate our *in vivo* activation by monitoring DC migration using adoptively transferred bioluminescent DCs (Luc-DCs) that bear the TRIGIR compound.

Further, to confirm that the migrating cells were presenting antigens and further priming adaptive immune cells^18, 19^, we performed RNA analysis on the target lymph-node. Reported herein is a general procedure where adoptively transferred immune cells can be remotely activated using a UV light source. Though this methodology calls for a TLR2/6 bearing cell type and has limited tissue penetration of UV light used to activate the cells, it may find use in controlling activation of skin or subcutaneous DCs and for studying effects of inflammation within different spatiotemporal parameters. We expect that improvements in both optogenetic techniques, longer wavelength photo-cages, and light delivery methods will help expand the technique to answer many different immunological questions.

## Results

### Cell labeling with NPPOC-Pam_2_CSK_4_

Previous work from our lab showed that photo-caging of the N-terminus of the TLR2/6 agonist, Pam_2_CSK_4_^20^, can inhibit its activity to activate TLR2/6. Upon light exposure and subsequent uncaging of the N-terminus, TLR2/6 is activated by the TRIGIR compound. The intercalation of the TRIGIR compound’s palmityl chains^21^ on the TLR2 of DCs allows labelling of the agonists to quiescent innate immune cells without activating TLR2/6. These labelled cells can then be used in adoptive transfer experiments to achieve remote control of inflammatory processes *via* TLR2.

We sought to adapt this technique *in vivo* by labeling cells, performing subcutaneous injection and then activating of the cells in their local environment. As the agonist stays co-localized, we can have the spatial control of agonist presentation and immune cell activation^17^.

**Figure 1:**
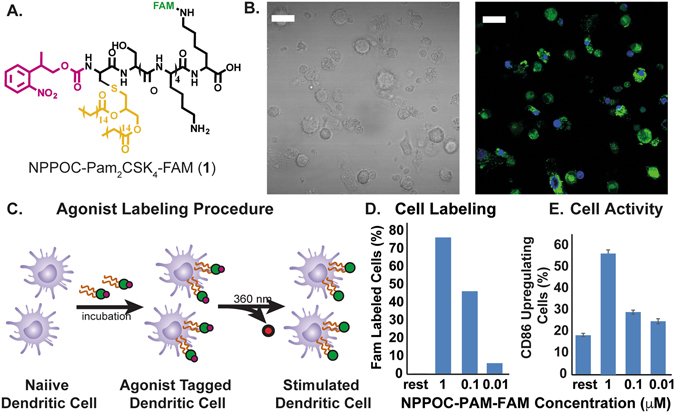
(**A**) Structure of photo-caged TLR 2/6 agonist NPPOC-Pam_2_CSK_4_ (**1, NPPOC-Pam-FAM**) with fluorescein tag, (**B**) bright field and fluorescent microscopic image of labeled DCs (green-**1**, blue-DAPI, scale bar 10 μm), (**C**) NPPOC-Pam-FAM labeling procedure, (**D**) Efficiency of agonist labeling, (**E**) background CD86 upregulation induced by labeling DCs.

In initial experiments, we observed that high concentration of the TRIGIR compound, NPPOC- Pam_2_CSK_4_ (**1**, Fig. 1A), incubation overnight resulted in higher amount of labeling of the agonist (Fig. 1D). However, this also resulted in higher background activation of the cells (Fig. 1E). Therefore, labeling the primary DCs, harvested from transgenic luciferase expressing mice, at 0.1 μM (Fig. 1C) showed both good labeling and did not elicit a background immune response (Fig. 1D,E).

### Photo-activation of transferred dendritic cells

**Figure 2:**
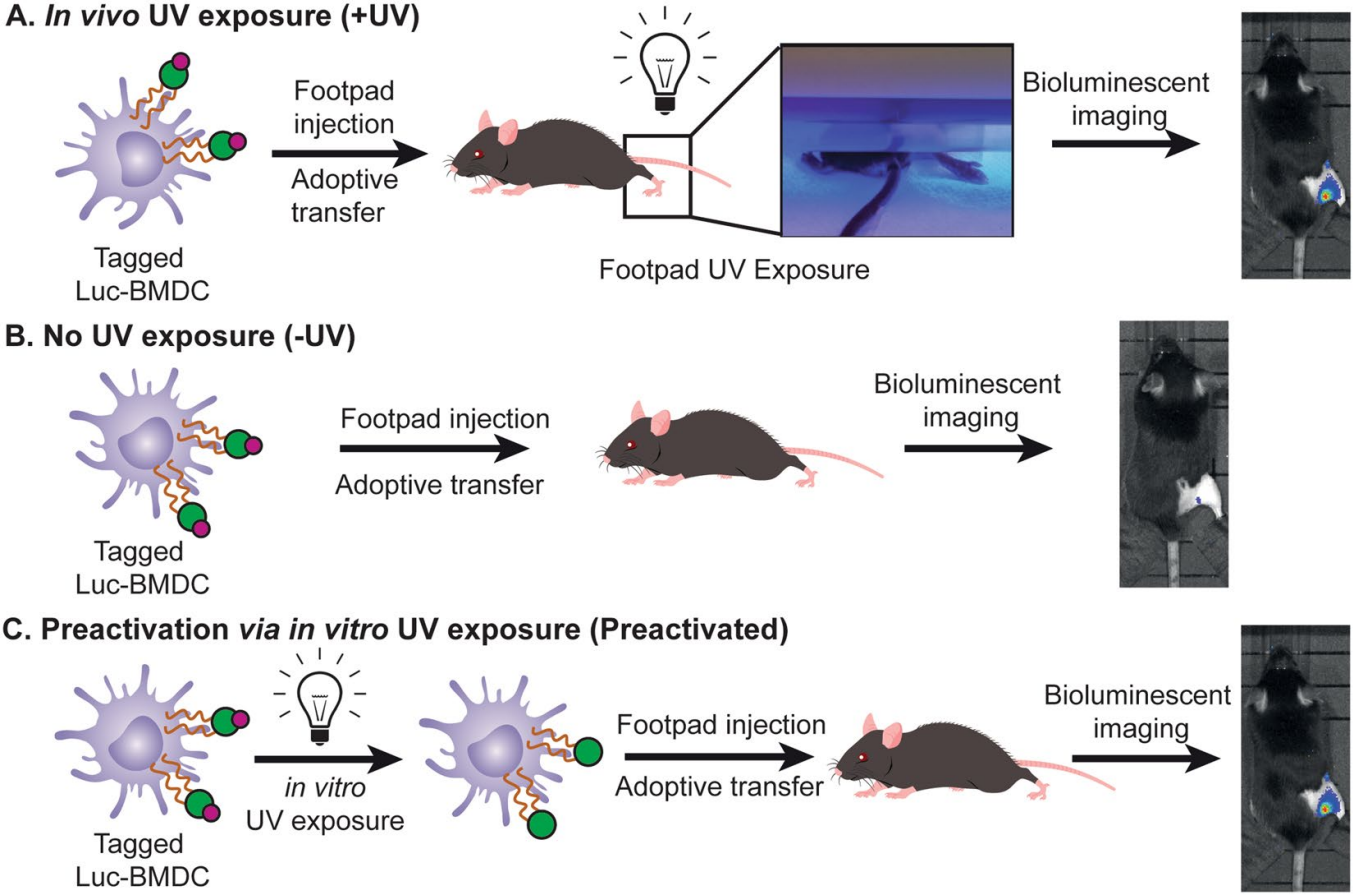
TRIGIR DC adoptive transfer procedures for (**A**) footpad UV irradiated mouse following adoptive transfer, (**B**) mouse with no UV irradiation, and (**C**) mouse injected with pre-irradiated tagged DCs.

Before adoptive transfer, the DCs were incubated with **1** over-night. The cells were then washed to remove excess **1** in the supernatant. The labeled cells were then injected into the footpad of mouse at 1 million cells/30 μL for the mice. To activate the cells with light, the injected footpad of mice was then irradiated with 360 nm light (15 W) for 15 mins (Figure SI 6). To determine the limit of activity due to the limit of UV light tissue penetration, we irradiated labelled cells with 360 nm light for 15 min *in vitro* before injection. This experiment served as a “pre-activated” control and served as an upper limit for what might be achieved with photo-activated DCs *in vivo*. During the imaging process, following previously reported procedures^22^, we blocked the bioluminescence occurring from the injected foot with black tape to enhance the signal from the popliteal lymph node (Fig. 2).

**Figure 3:**
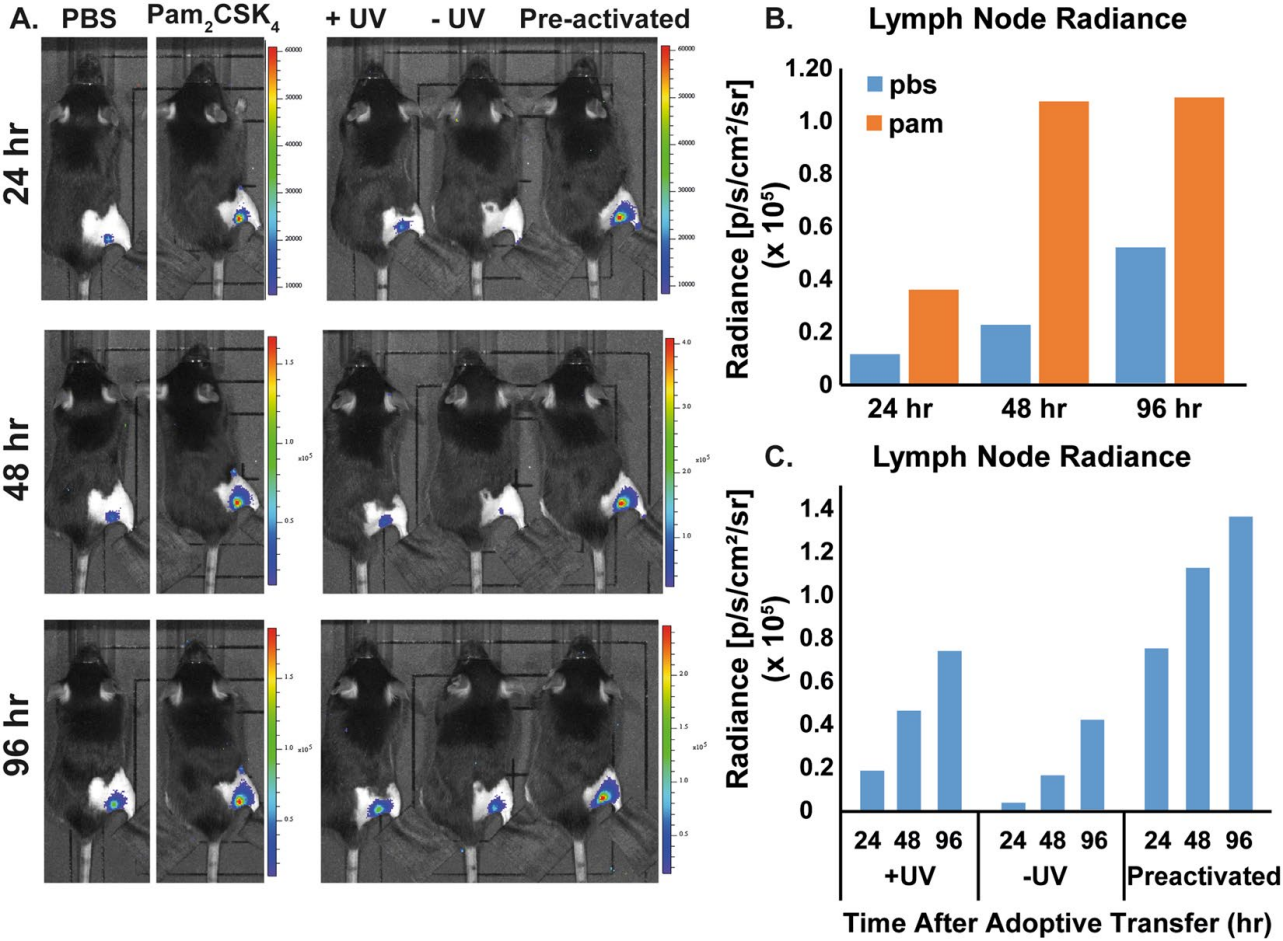
Bioluminescent image of mice taken every 24 h, over 96 h. (**A**) Control mice include a set of 6 mice injected with Luc-DCs preconditioned with Pam_2_CSK_4_, and a set of 6 mice injected with Luc-DCs with no preconditioning. The test set includes a set of 6 mice with TRIGIR labeled Luc-DCs followed by light exposure (+UV), one with no light exposure (−UV), and a mouse injected with TRIGIR labeled Luc-DCs exposed to light before footpad injection (pre-activated). (**B**) Radiance of lymph nodes of non-stimulated Luc-DCs and Pam_2_CSK_4_ stimulated Luc-DCs transferred mice. (**C**) Radiance of lymph nodes of irradiated, non-irradiated, and pre-activated mice. Data taken from representative group.

To understand the activity of mature DCs, we compared the migration of the Pam_2_CSK_4_ stimulated Luc-DCs and non-stimulated Luc-DCs that were adoptively transferred into the footpad of a mouse over a period of 96 hrs. We found the Pam_2_CSK_4_ stimulated Luc-DCs migrate faster than the unstimulated Luc-DCs, where we observed migration activity as early as 24 h in Pam_2_CSK_4_ stimulated Luc-DCs with a slow migration, over 96 hrs, of the unstimulated DCs into the draining lymph node at later time points (Fig. 3A,B). Because activation of dendritic cells leads to upregulation of cell surface receptors that aid in the migration and translocation of DCs into the lymph node^23^, we theorize that a shorter time is required for the activated cell to migrate into the lymph node compared to the unstimulated DCs.

We sought to determine if light-activation of TLR2 *via* **1** *in vivo* recapitulated the migration of activated DCs. We imaged the migration of the Luc-DC in mice whose footpads were exposed to UV light (+UV) or not exposed to UV (−UV). Following the trends seen in the Pam_2_CSK_4_ stimulated cells, the footpads which were directly exposed to UV showed migration of Luc-DCs into the popliteal lymph node much sooner than that of the non-exposed footpads (Fig. 3A,C). Additionally, the cells that were exposed to UV migrate at a similar rate as the cells that were photo-activated before being transferred into a mouse. From this data, we conclude that TRIGIR labelled cells can be activated with light in a non-invasive manner and recapitulate the timing and quantity of their migration to the lymph node.

### Confirmation of systemic activation *via* RNA analysis of popliteal lymph node

To further confirm the inflammatory state of TRIGIR activated DCs *in vivo* by light, we harvested popliteal lymph nodes from the mice and analyzed the RNA levels. This measurement also helped us determine if the activated DCs were enacting their antigen presenting role. If the cells were activated following light exposure, the migrated cells will elicit a systemic response as recruitment and maturation of adaptive immune cells occurs in the lymph node. We harvested lymph nodes from both light irradiated and non-irradiated animals which all contained TRIGIR-labeled DCs identical to our previous experiments. To determine differences, we plotted the changes as a relative fold-change of the from irradiated:non-irradiated at each time point. Using this measurement, we determined how irradiation and TLR stimulation changed activity in the lymph node.

**Figure 4:**
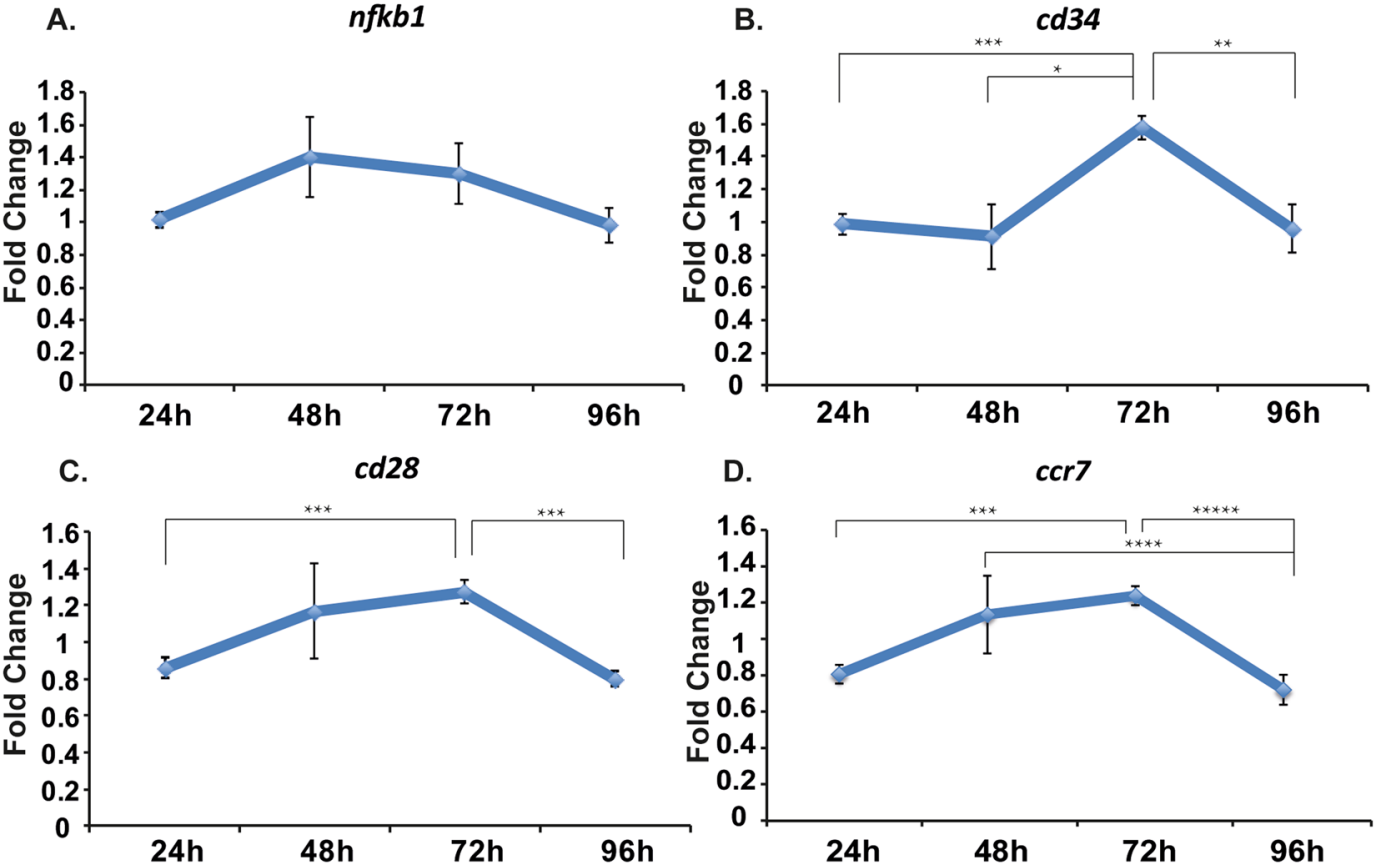
Change in gene profile in harvested lymph node of tested mice. Fold change determined by the ratio of UV irradiated and non-irradiated mice at each time point (n = 6) of *nfkb1* (**A**), *cd34* (**B**), *cd28* (**C**), and *ccr7* (**D**). Data taken from six individual mice. *p < 0.0056, **p < 0.0028, ***p < 0.0005, ****p < 0.0008, *****p < 0.035.

First, we observed that upon TRIGIR activation, there is a gradual increase in *ccr7* which is upregulated by immune cells that enter the lymph node through recognition of CCL19 and CCL21 on the lymph node (Fig. 4D)^24–26^. From this we conclude there are more *ccr7* producing cells recruited into the lymph node. These cells are likely the TRIGIR activated dendritic cells which we observed migrate to the lymph node as well as T cells that have been recruited into the lymph node within the first 72 h after UV exposure as a result of DC activation.

We saw further evidence for T cell recruitment upon TRIGIR activation with an increase of *cd34* and *cd28* within the same time period. CD34 is required for T cells to enter the lymph node while blocking DC migration into the lymph node^27^. The downregulation of *cd34* at early time points matches the increased migration of the stimulated DCs from the footpad into the lymph node (Fig. 4B). The gradual increase suggests the increase of T cell trafficking into the lymph node and decrease in DC migration from the footpad.

Similar to the *cd34* trends, we saw a gradual increase in *cd28*, a T cell receptor that recognizes CD80 and CD86^28^, reaching a maximum after 72hrs (Fig. 4C). This gradual rise indicates the increase in T cell population in the popliteal lymph node. These trends follow known T cell maturation and migration following mature DC contact in the lymph node^29^.

In comparison, there is a general upregulation of *nfkb1* ^30, 31^ starting as early as 48 hours, which could be due to the inflammatory signaling from the activated DCs that have migrated into the popliteal lymph node (Fig. 4A).

## Discussion

With our method of *in vivo* photo-activation of immune cells, we delivered a photo-caged, TRIGIR agonist and activated it in a non-invasive manner with light. Using the TRIGIR method of tagging cells, we can overcome the limitation of spatial control of soluble agonists as well as site-specific cell delivery. Compared to conventional adoptive transfer methods that require activation of cells prior to transfer to the animal our method allows for less steps in preparation of the transferred cells and controls when the cells will be activated following adoptive transfer. In addition to temporal control of cell activation, this method offers for the potential of light dosage dependent mitigation of inflammatory signals where longer irradiation times would activate more cells, allowing for sustained activation without the increasing inflammatory response.

This method can also be applied to a variety of cells to induce different responses to TLR2/6 activation. Because TRIGIR is cell specific, but requires labeling, it is compatible with many different primary cell types that can be adoptively transferred. By changing the types of cells and cell populations, one can dissect not only autocrine signaling, but also paracrine signaling following light activation of cell subsets. The technique will not limit researchers to adoptive transfer in the footpad but can create a depot of tagged, subcutaneous cells placed close to an area of interest and gain spatial and temporal control of elicited cellular response. We offer the clear caveat that current photo-activation methods will limit this method to dermal or subcutaneous activation of innate immune cells. Our data suggest that this technique will give researchers the potential to customize an innate cellular response depending on the target disease or immunological model. In conclusion, we present a method for light activation of adoptively transferred cells *via* TLR2/6. This technique presents a unique way to answer spatial and temporal questions about the innate immune response.

## Methods

All animal studies and mice maintenance were carried out in accordance with relevant gidelines and regulations approved by the Institutional Animal Care and Use Committee at University of California, Irvine (IACUC #2012-3048).

### Bone Marrow-Derived Dendritic Cell Harvest and Culture

Bone marrow-derived dendritic cells (BMDCs) were harvested from 6-week-old B6;FVB-*Ptprc* ^*a*^ Tg(CAG-luc,-GFP)L2G85Chco *Thy1* ^*a*^/J mice (Jackson Laboratory). Femur bones were removed from mice and the bone marrow was extracted into PBS buffer and pelleted. ACK Lysing Buffer (3 mL, Lonza) was added to the cell pellet and incubated for 2 min at RT. PBS buffer (13 mL) was then added to the cell suspension, and the cell solution was centrifuged at 300 RCF for 10 min at RT. Thereafter, the cell pellet was resuspended in BMDC complete media composed of RPMI 1640, 10% heat inactivated FBS, 20 ng/mL granulocyte-macrophage colony-stimulating factor (GM-CSF), 2 mM L-glutamine (Life Technologies), 10,000 U/mL penicillin, 10 mg/mL streptomycin, 25 μg/mL amphotericin B, and 50 μM beta-mercaptoethanol. Harvested cells were plated at 1 × 10^6^ cells/mL in 100 mm petri dishes (10 mL total media) and incubated at 37 °C in a CO_2_ incubator (day 0 of cell culture). On day 3, 10 mL of fresh BMDC primary media was added to each petri dish. On day 5, BMDCs were released and plated in 24-well plates at 5 × 10^5^ cells/mL for cell surface marker activation, cytokine profile flow cytometry experiments.

### General Procedure for Flow Cytometry for Cell Surface Marker Upregulation

BMDCs were incubated in individual wells with each agonist (9:1 BMDC:agonist) in 0.5 mL culture media for 18 h at 37 °C with 5% CO_2_. The cells were released from the plate and centrifuged at 2500 RPM at 4 °C for 10 min. The cell pellet was resuspended in cold FACS (composed of PBS (1x), 10% FBS, and 0.1% sodium azide) buffer (100 μL) and incubated with CD16/32 FcR blocking antibodies (1.0 μg/1 × 10^6^ cells) on ice for 10 min. The cell suspension was pelleted and the supernatant was removed. The cell pellet was resuspended in cold FACS buffer (100 μL) and incubated with PE-CD86 (1.0 μg/1 × 10^6^ cells) on ice and removed from light for 30 min. Each sample was washed twice with 300 μL cold fluorescence-activated cell sorting (FACS) buffer. The dendritic cells were resuspended in cold FACS buffer (150 μL) and kept on ice until being loaded onto the flow cytometer.

### General Procedure for Cell Labeling

BMDCs were incubated at 3 × 10^6^ cells in 2 mL of media in a 6 well cell culture plate with the addition of NPPOC-Pam-FAM at 100 nM overnight at 37 °C with 5% CO_2_. Following incubation, the cells were collected in 15 mL conical tubes and rinsed with PBS 5 times. After the final rinse, the cells were counted and resuspended in PBS at a final cell concentration of 1 million cells/30 μL of PBS.

For the *ex vivo* UV exposed cells, the labeled cells were deprotected with 365 nm light following the last rinse, counted, and resuspended in PBS at a final cell concentration of 1 million cells/30 μL of PBS.

### General Procedure for Adoptive Transfer

Labeled Luc-BMDCs were adoptively transferred *via* subcutaneous injection in the footpad of a C57/BL6J (Jackson Lab) mouse. The labeled cells were loaded into a syringe (10 cc, insulin syringe) at 1 million cells/ 30 μL of PBS. UV exposed mice were put under isofluorane (2% in 1 L/min O_2_) and exposed to UV light (UVP 95-01300-01 BL-15 long wave UV lamp, 15 W) for 15 mins.

### IVIS Imaging Procedure

Luciferin was injected into each mouse (15 mg/mL in sterile PBS, 10 uL/g/mouse) *via* intraperitoneal injection. After 10 mins following the luciferin injection, the mice were anesthetized with isoflurane (2% in 1 L/min O_2_). Before taking images the injected foot was taped with black athletic tape and black electrical tape (3 M) to enhance the bioluminescent signal from the lymph node. Images were analyzed using Living Image Software.

### Lymph Node Tissue Harvest and RNA Extraction

Popliteal lymph nodes were harvest following each designated time point and suspended in RNAlater solution for up to 2 weeks. The harvested RNA was homogenized with prefilled 2 mL, 1.5 mm Zirconium bead tubes at 250 G for 90 secs. The homogenized tissue solution was extracted for RNA following the procedures for RNeasy Mini Kit (Qiagen). cDNA was reverse transcribed using the extracted RNA (KIT). Murine *ccr7, cd34, cd28, nfkb1* expression was quantified using Maxima SYBR Green/ROX qPCR Master Mix (Thermo Fisher) in the ABI 7300 detection system (Applied Biosystems). GAPDH gene expression was measured as endogenous reference. The relative fold change was calculated following the 2^-ddCT method^32^. Fold change was normalized to the average of non-irradiated mice (non treated group) and UV mice (treated group) of triplicate of 6 different mice in each group.

## Electronic supplementary material

Supplementary Information
