# Supplementary material for: Light Guided In-vivo Activation of Innate Immune Cells with Photocaged TLR 2/6 Agonist
Source: Sci Rep. 2017 Aug 14;7:8074. doi: 10.1038/s41598-017-08520-x (PMC5556111; doi:10.1038/s41598-017-08520-x)
Supplement: Supplementary file 1 — Supplementary Information [file 41598_2017_8520_MOESM1_ESM.pdf]

Supporting Information

Light Guided *In-vivo* Activation of Innate Immune Cells with Photocaged TLR 2/6 Agonist

Keun Ah Ryu, Bethany McGonnigal, Troy Moore, Tawnya Kargupta, Rock J. Mancini, Aaron P. Esser-Kahn

Dept. of Chemistry, University of California, Irvine, Irvine, CA 92697, USA.

Contents

Materials and Methods 2
Supplementary Figures 2-8

Materials and Methods

Unless otherwise noted, all reagents were purchased from Sigma-Aldrich and used as received. D-Luciferin (Potassium salt) was purchased from Gold Biotechnology. Antibodies were purchased from BioLegend. BD Cytofix/Cytoperm Kit for intracellular cytokine flow cytometry and GolgiPlug were purchased from BD Biosciences. Automated solid-phase peptide synthesis was carried out by using a PS3TM Peptide Synthesizer (Protein Technologies, Inc.). Analytical reversed-phase HPLC was performed in an Agilent Zorbax SB-C18 column (50 mm4.6 mm) with a gradient of 5–100% CH_3CN in H_2O with 0.1% TFA and a flow of 1.0 mL/ min over 20 min. UV Vis was measured on Thermo Scientific Nano Drop. Flow cytometry data was acquired using BD Accuri C6 Flow Cytometer and analyzed using the BD Accuri C6 software. RAW-Blue absorbance was measured on a Bio-Tek Quant microplate spectrophotometer MQX200. Microscopy images were obtained on Zeiss 780 confocal microscope in the UCI Optical Biology Core. IVIS images were taken on an IVIS Lumina (Xenogen) system. Lymph node tissue work up was done with Genesee Scientific Bead Bug, RNA extraction was done with Qiagen RNeasy mini kit. qPCR was performed on ABI 7300 with Maxima Sybr green (thermo-fisher) and primers ordered from IDT technologies. C57/BL6J (wild type), B6;FVB-*Ptprc*^{a} Tg(CAG-luc,-GFP)L2G85Chco *Thy1*^{a}/J (lucfiferase expressing C57/BL6J) were purchased from Jackson Laboratories and allowed to equilibrate for a minimum of 48 h. All animal studies and mice maintenance were approved by the Institutional Animal Care and Use Committee (IACUC #2012-3048).

Agonist Synthesis Procedures

See preparation in Mancini *et. al.*[1]

Supplementary Figures

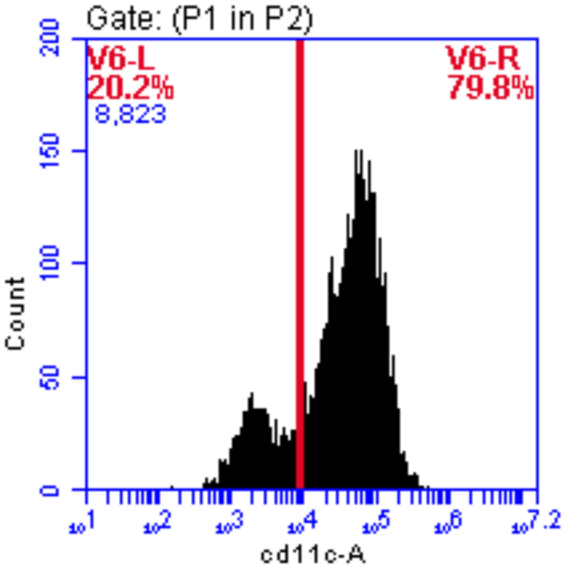

SI 1. CD11c^{+} BMDC purity within primary BMDC cell culture following procedures discussed in methods and materials measured *via* flow cytometry.

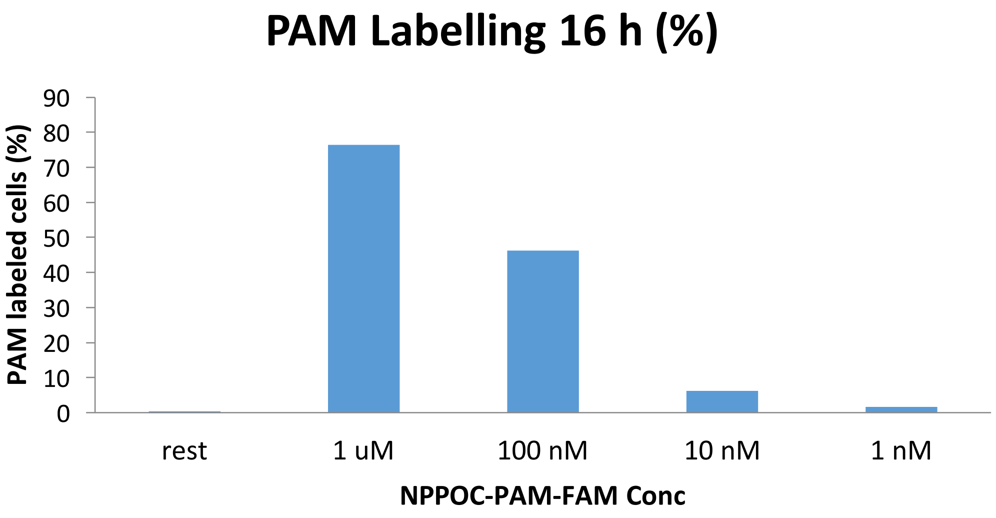

SI 2. NPPOC-Pam-FAM labeling measured *via* flow cytometry with changing NPPOC-Pam-FAM concentrations. DCs incubated with NPPOC-Pam-Fam overnight (experiment run in duplicate).

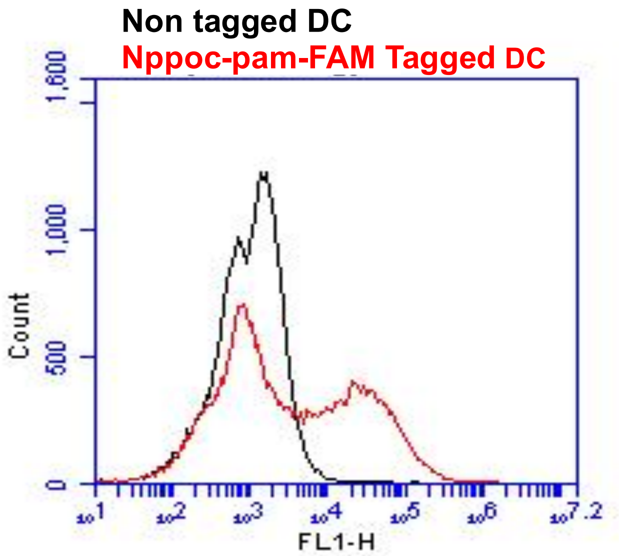

SI 3. Flow cytometry plot of non-tagged DC (black) and NPPOC-Pam-FAM tagged DC (red).

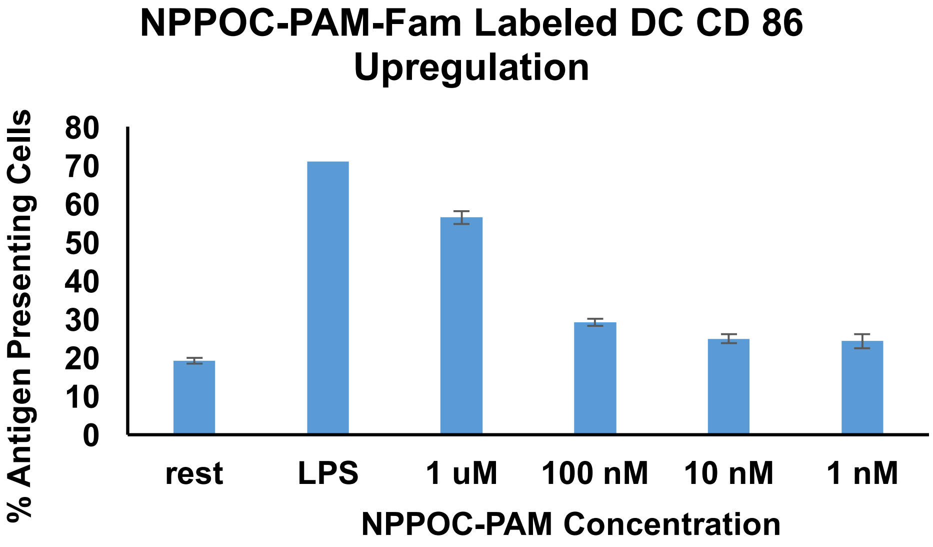

SI 4. To measure the background activation from the labeling procedure alone (no irradiation), CD86 presentation of DCs tagged with NPPOC-Pam-fam over 16 hrs was measured. Each result is from three independent experiments where $p<0.046$ for all points.

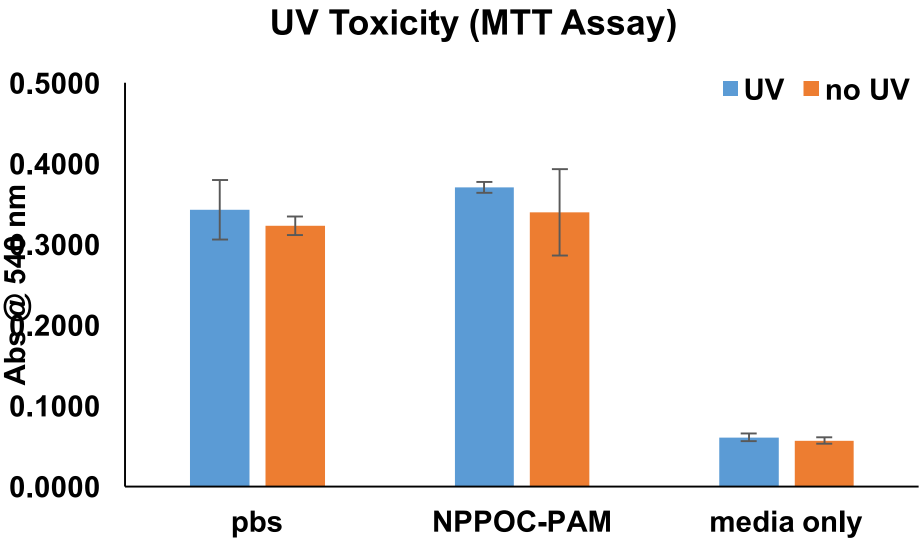

SI 5. Viability of labeled and irradiated cells measured with MTT assay. Each result is from five independent experiments where $p<0.42$.

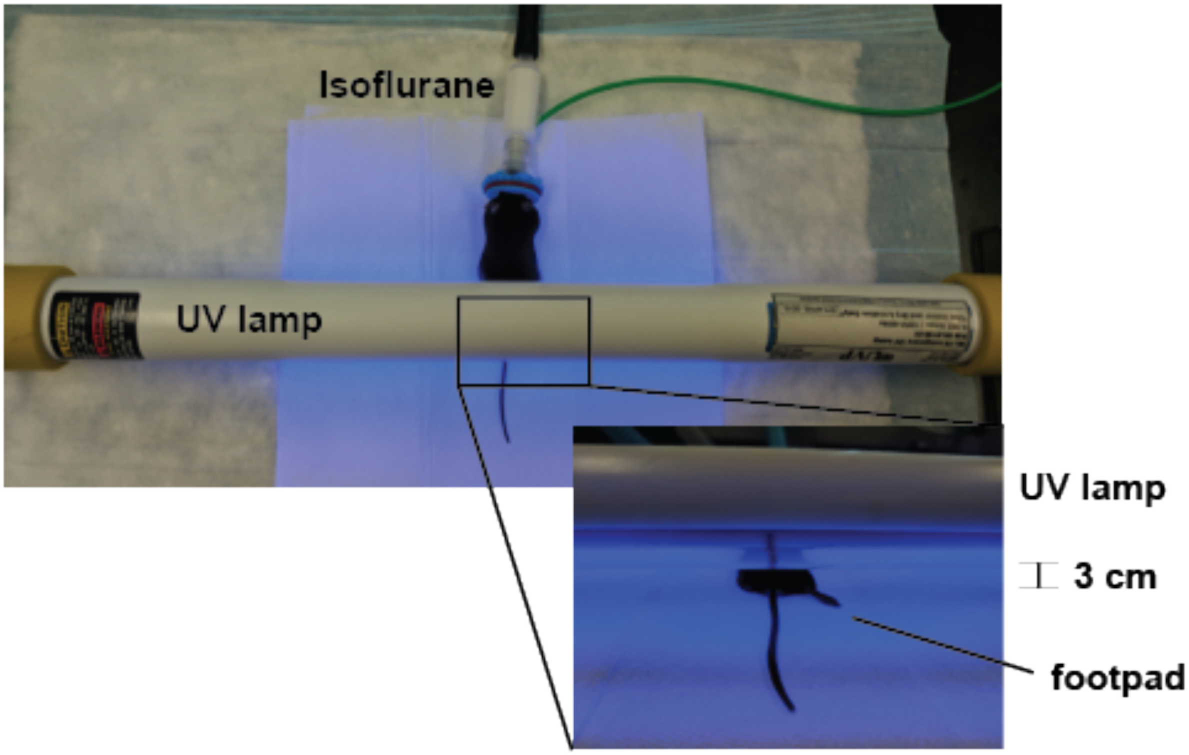

SI 6. Mouse irradiation set up

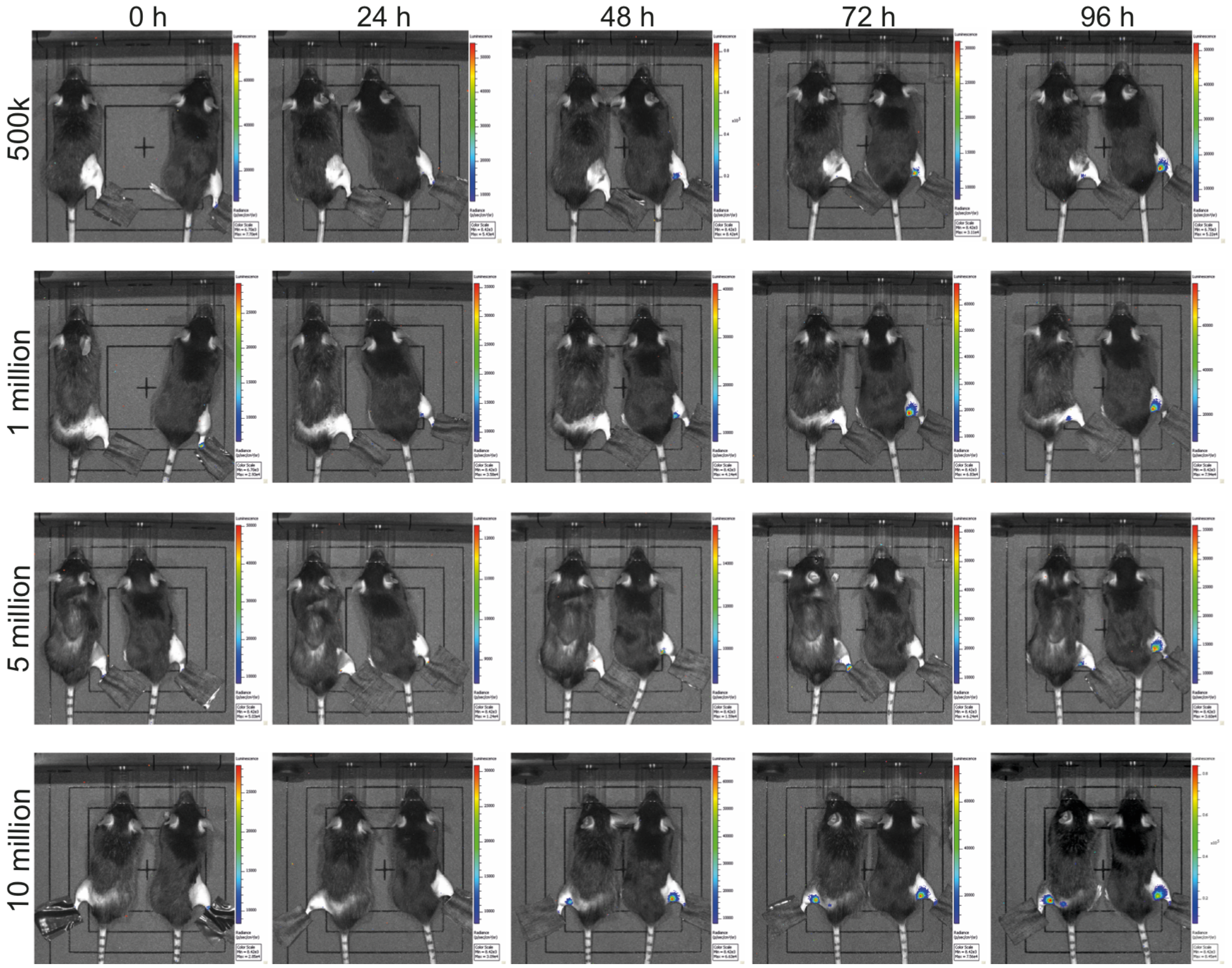

SI 7. Optimization of cell concentration of adoptive transfer (n=2). Mice injected with $1x10^7$cells showed migration as early as 48 hrs. Migration was similar for concentrations of $5x10^5$, $1x10^6$, and $5x10^6$, however we chose to perform all adoptive transfer assays at $1x10^6$, as previous studies reported loss in efficiency of cell expansion and activity of footpad injected DCs at concentrations higher than $2x10^6$.[3] Luminescence measured in radiance ($p/s/cm^2/sr$).

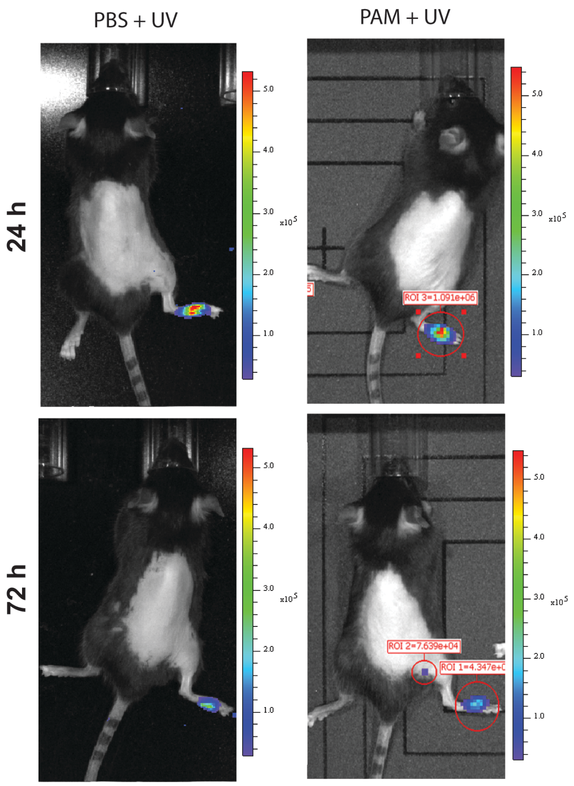

SI 8. No light dependent migration of control of UV irradiated non-stimulated BMDC (PBS) following footpad injection over 72 hrs was observed. Luminescence measured in radiance (p/s/cm^2/sr).

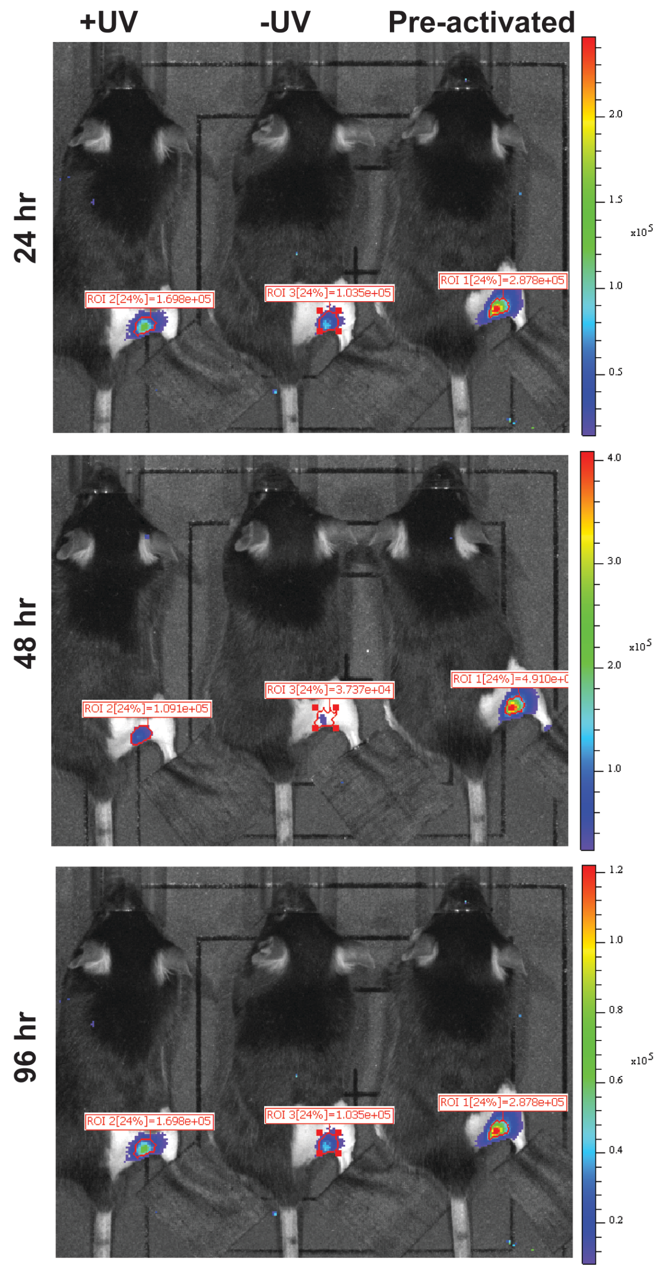

SI 9. ROI of each mouse was measured using automated ROI tool in living image program. Luminescence measured in radiance (p/s/cm^2/sr).

nfkb1	24 h	48 h	72 h	96 h
24 h		0.055	0.065	0.66
48 h			0.58	0.053
72 h				0.065
96 h				

cd34	24 h	48 h	72 h	96 h
24 h		0.57	0.0005	0.79
48 h			0.0056	0.75
72 h				0.0028
96 h				

cd28	24 h	48 h	72 h	96 h
24 h		0.11	0.001	0.22
48 h			0.53	0.072
72 h				0.0004
96 h				

ccr7	24 h	48 h	72 h	96 h
24 h		0.061	0.0005	0.2
48 h			0.46	0.035
72 h				0.0008
96 h				

SI 10. Table of statistical significance (p<) of RNA expression fold change (Figure 4).

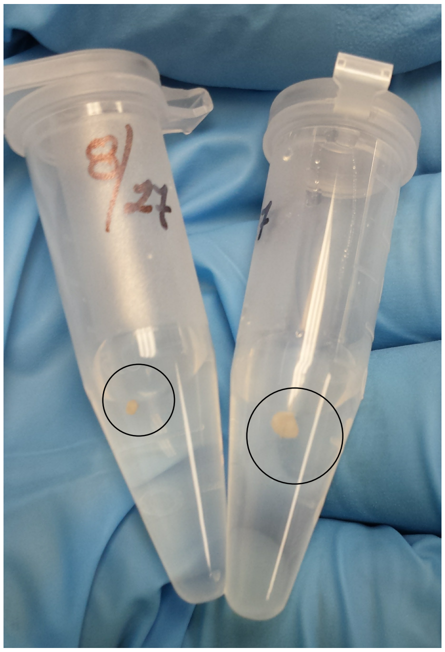

SI 11. Popliteal lymph node of non-injected footpad (left) and DC injected side popliteal lymph node (right), harvested 96 h after adoptive transfer

REFERENCES

1. Mancini, R. J.; Stutts, L.; Moore, T.; Esser-Kahn, A. P., Controlling the Origins of Inflammation with a Photoactive Lipopeptide Immunopotentiator. *Angew. Chemie. Int. Ed.* **2015,** *54* (20), 5962–5965.
2. Livak, K.; Schmittgen, T., Analysis of relative gene expression data using real-time quantitative PCR and the 2(-Delta Delta C(T)) Method. *Methods* **2001,** *25* (4), 402-408.
3. Martín-Fontecha, A.; Sebastiani, S.; Höpken, U. E.; Uguccioni, M.; Lipp, M.; Lanzavecchia, A.; Sallusto, F., Regulation of Dendritic Cell Migration to the Draining Lymph Node. *The Journal of Experimental Medicine* **2003,** *198* (4), 615-621.
